# *ABCB1* C3435T polymorphism and the lipid-lowering response in hypercholesterolemic patients on statins: a meta-analysis

**DOI:** 10.1186/s12944-015-0114-2

**Published:** 2015-10-06

**Authors:** Jia Su, Hongyu Xu, Jun Yang, Qinglin Yu, Shujun Yang, Jianjiang Zhang, Qi Yao, Yunyun Zhu, Yuan Luo, Lindan Ji, Yibo Zheng, Jingbo Yu

**Affiliations:** Department of Gerontology, Ningbo No.1 Hospital, Ningbo, Zhejiang Province 315010 People’s Republic of China; Department of Traditional Chinese Internal Medicine, Ningbo No.1 Hospital, Ningbo, Zhejiang Province People’s Republic of China; Department of Hematology, Ningbo No.1 Hospital, Ningbo, Zhejiang Province People’s Republic of China; Department of Biochemistry, School of Medicine, Ningbo University, Ningbo, Zhejiang Province People’s Republic of China

**Keywords:** *ABCB1*, Polymorphism, Statin, Serum lipid level, Myopathy, Meta-analysis

## Abstract

**Background:**

A number of researches have evaluated the association between the *ABCB1* polymorphism and the lipid-lowering response of statins, but the results have been inconclusive. To examine the lipid-lowering efficacy and safety associated with the *ABCB1* C3435T polymorphism in hypercholesterolemic patients receiving statin, all available studies were included in this meta-analysis.

**Methods:**

A systematic search for eligible studies in the Cochrane library database, Scopus and PubMed was performed. Articles meeting the inclusion criteria were comprehensively reviewed, and the available data were accumulated by the meta-analysis.

**Results:**

The results indicated that the comparisons of CC+CT vs. TT were associated with a significant elevation of the serum HDL-C levels after statin treatment (CC+CT vs. TT: MD, 2.46; 95 % CI, 0.36 to 4.55; *P* = 0.02), and the *ABCB1* C3435T variant in homozygotes was correlated with decreases in LDL-C (CC vs. TT: MD, 2.29; 95 % CI, 0.37 to 4.20; *P* = 0.02) as well as TC (CC vs. TT: MD, 3.05; 95 % CI, 0.58 to 5.53; *P* = 0.02) in patients treated with statin. However, we did not observe a significant association in the TG group or an association between other genetic models serum lipid parameters. In addition, statin treatment more than 5 months led to a higher risk of muscle toxicity.

**Conclusions:**

The evidence from the meta-analysis demonstrated that the *ABCB1* C3435T polymorphism may represent a pharmacogenomic biomarker for predicting treatment outcomes in patients on statins and that statin treatment for more than 5 months can increase the risk of myopathy.

## Introduction

Cardiovascular diseases are the leading cause of death worldwide, and atherosclerosis due to lipid metabolism is one of the main determinants of cardiovascular risk [1]. Statins, which inhibit 3-hydroxy-3-methylglutaryl co-enzyme A reductase (HMG-CoA reductase) are effective at reducing atherosclerosis and cardiovascular risks in clinical practice by lowering the levels low-density lipoprotein cholesterol (LDL-C) and total triglycerides (TG) [2–4]. However, the pharmacodynamic response to statins varies greatly among patients [5]. Although the mechanisms have not been fully clarified, genetic polymorphisms may play an important role in individual susceptibility to drug response, including the *ABCB1* C3435T (rs1045642) genetic variant.

The *ABCB1* (adenosine triphosphate -binding cassette, sub-family B, member 1) gene, which also called multidrug resistance 1 gene (MDR1), encodes the intestinal efflux transporter P-glycoprotein, which has been associated with the transport of cellular lipids and drugs [6]. More than 50 single-nucleotide polymorphisms (SNP) in the *ABCB1* gene, located at 7, p21-21.1 [7], have been described in the literature. Among them, *ABCB1* C3435T (rs1045642) is the extensively investigated, and many studies have shown that the *ABCB1* C3435T genotype influences ability of P-glycoprotein to direct the absorption of statins [8].

Both the lipid-lowering response of statin and their safety has been investigated. Kajinamietal. [9] first analyzed the effect of the C3435T polymorphism on clinical outcomes in patients receiving atorvastatin and found that the C3435T polymorphism was significantly and independently related to a smaller reduction in LDL-C and a larger increase in high-density lipoprotein cholesterol (HDL-C) relative to variant allele carriers in a gender-specific manner. One study [10] reported that the *ABCB1* C3435T polymorphism differed statistically between the ADR (muscle toxicity) and non-ADR groups treated with simvastatin. However, the results from different studies [9–16] have been inconsistent. Therefore, we performed a meta-analysis to determine the relationship between the *ABCB1* C3435T polymorphism and the lipid-lowering efficacy and safety of statins.

## Methods

### Literature search

Three electronic databases (PubMed, Scopus and the Cochrane library) were comprehensively searched. The latest search was updated in February 2015 with the following terms: statin, *ABCB1, MDR1,* multidrug resistance, polymorphism, myopathy, myositis, and rhabdomyolysis. All the eligible studies were retrieved, and the bibliographies of the eligible studies and previous meta-analyses were checked for additional relevant articles.

### Inclusion criteria

Studies that meet the following criteria were included: (1) published in English, (2) case- control studies on lipid-lowering therapy, (3) the evaluation of the *ABCB1* C3435T polymorphism, the lipid-lowering efficacy and safety in patients receiving statin, (4) the availability of the genotype frequency in studied population, and (5) available data to calculate a mean difference (MD) or *P*-value with a 95 % confidence interval (CI).

### Data extraction

Two reviewers reviewed all studies carefully, extracted the data independently, and reached a consensus on all aspects. The following information was obtained from each study: the first author’s name, the publication date, the country of studied population, sex, age, follow-up period, treatment protocol, serum lipids parameters, myopathy incidence, statin, and gene information.

### Study outcomes

Two endpoints—the lipid-lowering efficacy and safety—were studied. The efficacy was evaluated by the change in TC (total cholesterol), TG, HDL-C, and LDL-C after statin treatment. The lipid-lowering safety in this study was reflected in the number of adverse effects of muscle toxicity, which comprised myalgia, myopathy, rhabdomyolysis or statin-induced elevations in serum Creatinekinase(CK) [17].

### Statistical analysis

The observed genotype frequencies in the controls were compared with the expected genotype frequencies using the Hardy-Weinberg equilibrium (HWE). The mean difference (MD) and 95 % confidence interval (CI) in each study was used to assess the strength of the association between the *ABCB1* C3435T polymorphism and lipid-lowering efficacy and safety in patients who received statin treatment. The pooled MDs were assessed for the dominant genetic model (CT+TT vs.CC), recessive genetic model (TT vs. CC+CT) and homozygote comparison (TT vs. CC) according to the method reported by Woolf [18]; significance was evaluated using the Z-test. Heterogeneity between studies was examined through the use of the *χ*2-based Q statistic test and was considered significant if *p* <0.1 [19]. The I^2^statistic was used to efficiently test for the heterogeneity [20]. If the effects appeared to be homogeneous, the fixed-effect method was used. Otherwise the random-effect model was adopted.

The heterogeneity was tested by subgroup analyses. Sensitivity analyses were performed to identify the potential influence of each study set on the pooled MDs by the sequential omission of individual studies. Publication bias was also detected by the funnel plot, and the symmetry of the plot distribution indicated the absence of publication bias [21]. All statistical tests were conducted using Review Manager (v.5.1, The Cochrane Collaboration), and were considered significant if the two-tailed *P* value was less than 0.05.

## Result

### Study characteristics

**Fig. 1:**
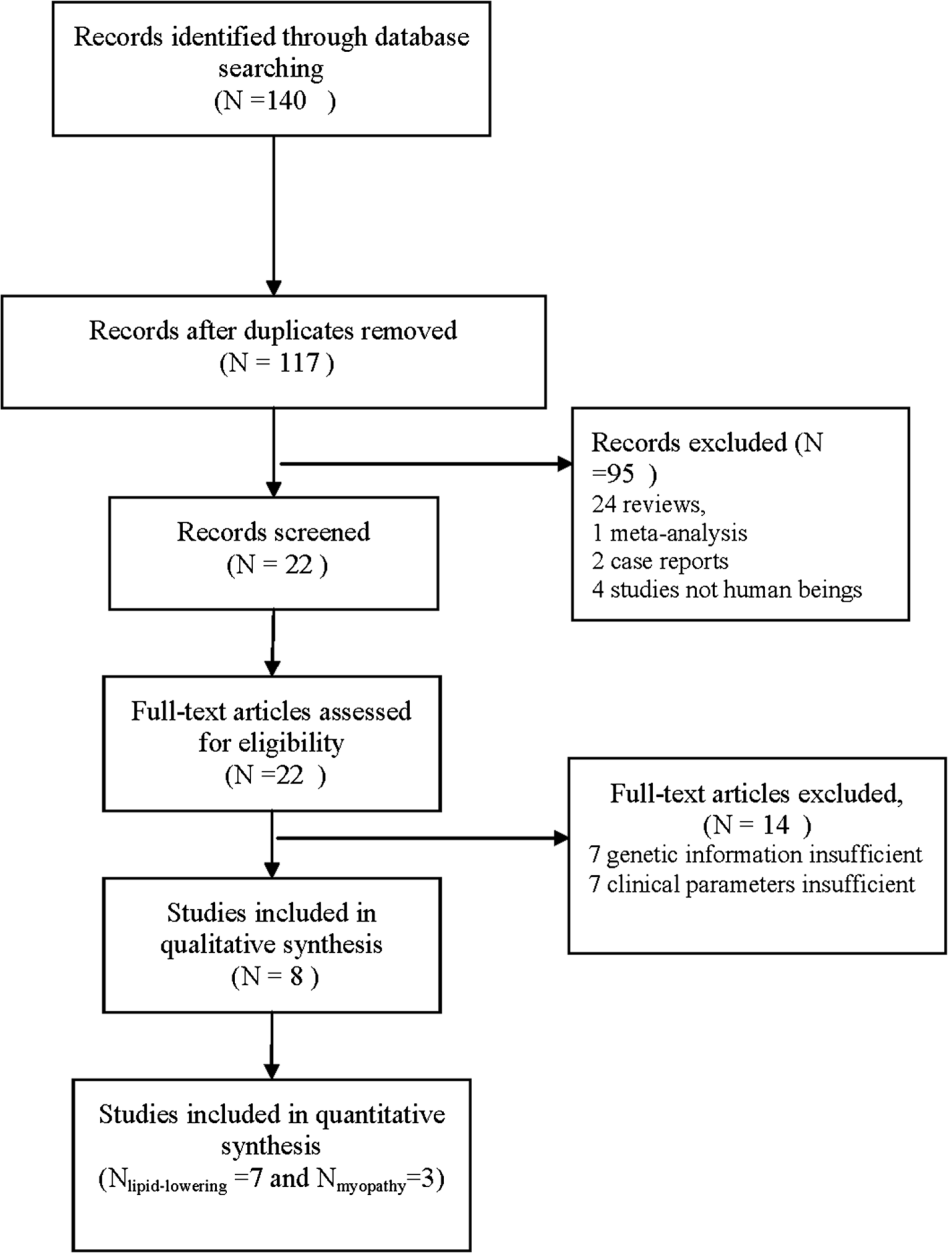
Flow diagram of the trial selection process

A total of 140 studies on the *ABCB1* C3435T polymorphism and the lipid-lowering efficacy and safety of statins were identified, and 23 replicated studies were excluded. Additionally, 24 reviews, 1 meta-analysis, 2 case reports and 4 studies not involving human beings were excluded. Meanwhile, 64 irrelevant studies were excluded after reviewing the title and abstract. Furthermore, seven studies were excluded because of their insufficient genetic information, and seven trials were excluded because the serum lipid parameters (TC, TG, HDL-C, and LDL-C) and the number of cases of myopathy were not available. Finally, eight studies, including seven studies (930 patients) of the lipid-lowering efficacy and three of statin-associated myopathy (58 cases and 239 controls), met the inclusion criteria (Fig. 1). The characteristics of the included studies are summarized in Table 1 and Table 2. All the studies were performed on a representative sample of the target group. Various statin treatment protocols were applied, including simvastatin 20 mg/day [10], atorvastatin 80 mg/day [15], atorvastatin arm 40 mg/day [14], and atorvastatin arm 10 mg/day [9, 12, 13], among others (atorvastatin, rosuvastatin or simvastatin) which we could not be determined precisely based on the original articles [11, 16]. The frequencies of the genotypes in each study (Tables 1 and 2) all followed the HWE.

**Table 1:** Main characteristics of studies included on lipid-lowering therapy in the meta-analysis

									Genotype			
First author	Year	Country	Population	Gende (male %)	Age	Follow-up period	Treatment protocal	Lipid	CC	CT	TT	HWE
Fiegenbaum [10]	2005	Brazil	Hypercholesterolemic patients	25.3	59.2 ± 10.7	6 months	simvastatin 20 mg/day	TC LDL-C HDL-C TG	20	45	32	0.570
Hoenig [15]	2011	Australia	Patients recruited with CAD, ischemic stroke, or CAD risk-equivalents	79	67.6 ± 10	6 weeks	atorvastatin 80 mg/day	TC LDL-C HDL-C TG	15	42	41	0.440
Kajinami m [9]	2004	USA	Hypercholesterolemia patients	100	58 ± 11	52 weeks	atorvastatin 10 mg/day	TC LDL-C HDL-C TG	49	101	56	0.793
Kajinami w [9]	2004	USA	Hypercholesterolemia patients	0	58 ± 11	52 weeks	atorvastatin 10 mg/day	TC LDL-C HDL-C TG	36	60	42	0.130
Rodrigues [13]	2005	Brazil	Hypercholesterolemia patients	40.6	59 ± 12	4 weeks	atorvastatin 10 mg/day	TC LDL-C HDL-C TG	19	36	14	0.684
Rosales [12]	2012	Chile	Hypercholesterolemia patients	62.7	56.4 ± 10.7	1 month	atorvastatin 10 mg/day	TC LDL-C HDL-C TG	60	68	14	0.404
Shabana [14]	2013	Egypt	Hypercholesterolemia patients	57	55.2 ± 9.9	4 weeks	atorvastatin 40 mg/day	TC LDL-C HDL-C TG	19	20	11	0.206
Salacka [11]	2014	Poland	NA	43	NA	2-83weeks	atorvastatin 10–20 mg/day, simvastatin 20–40 mg/day	TC HDL-C TG	25	65	40	0.878

*NA* Not applicable, *HWE* Hardy-Weinberg equilibrium, *TC* Total cholesterol, *LDL-C* Low-density-lipoprotein cholesterol, *HDL-C* High-density-lipoprotein cholesterol, *TG* Triglycerides

**Table 2:** Main characteristics and genotype of studies included on myopathy due to statin treatment in the meta-analysis

								Case			Control			
First author	Year	Country	Gende (male %)	Age	follow-up period	Treatment protocal	The definition of the case	CC	CT	TT	CC	CT	TT	HWE
Fiegenbaum [10]	2005	Brazil	25.3	59.2 ± 10.7	6 months	simvastatin 20 mg/d	Myalgia	5	10	0	21	46	32	0.557
Hoenig [15]	2011	Australia	79	67.6 ± 10	6 weeks	atorvastatin 80 mg/d	Myalgia	1	2	7	18	46	43	0.349
Ferrari [16]	2014	NA	39.4	NA	more than 5 months	atorvastatin, rosuvastatin or simvastatin	statin-induced elevations in serum CK of >3 × UNL	4	17	12	11	17	5	0.707

*NA* Not applicable

### Meta-analysis results

#### Association with lipid-lowering therapy

##### HDL-C

**Fig. 2:**
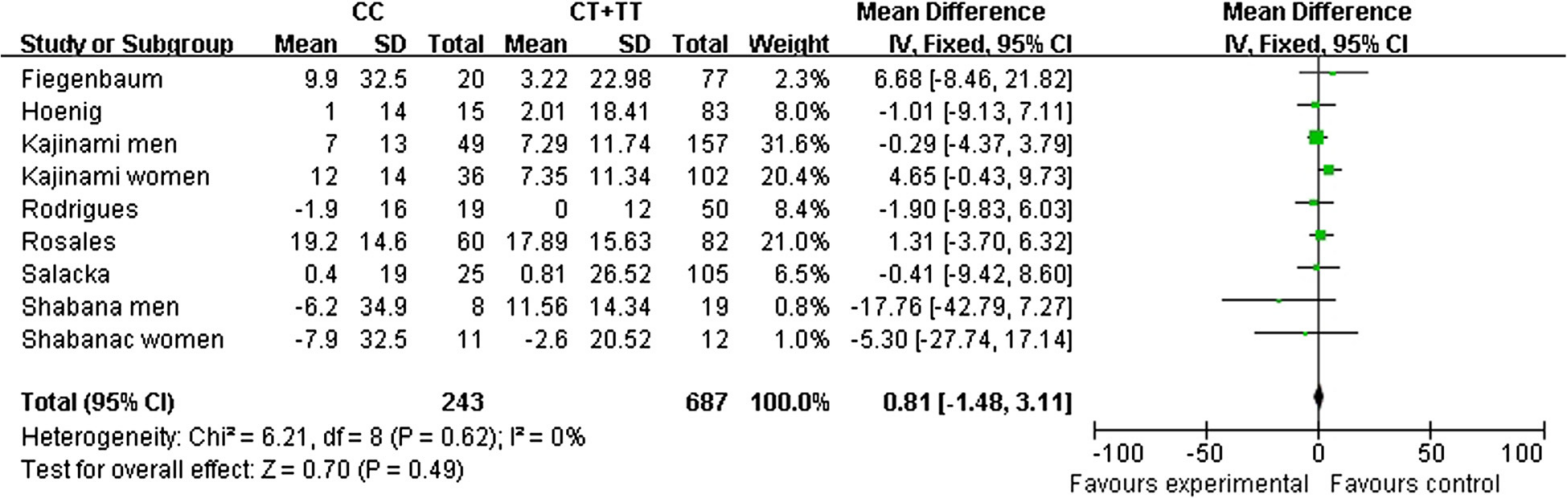
The association of the *ABCB1* C3435T polymorphism with the pooled fix-effects-based mean difference of the change in HDL-C after statin treatment. Comparison: CC vs. CT+TT

The comparison of CC+CT vs. TT was associated with a significant elevation in the serum HDL-C levels upon statin treatment (CC+CT vs. TT: MD, 2.46; 95 % CI, 0.36 to 4.55; *P* = 0.02; I^2^ = 22 %; Fig. 2 and Table 3). No significant relationship between the change in the serum HDL-C level and other genetic models of the *ABCB1* C3435T polymorphism were observed, and no heterogeneity was identified.

**Table 3:** The ABCB1 C3435T polymorphism on lipid-lowering therapy

	CC vs. CT+TT			CC+CT vs. TT			CC vs. TT		
Variables	OR (95 % CI)	P^a^	P	OR (95 % CI)	P^a^	P	OR (95 % CI)	P^a^	P
TC	1.36(−0.24,2.95)	0.64	0.09	1.33(−0.11,2.77)	0.45	0.07	2.29(0.37,4.20)	0.76	0.02
HDL-C	0.81(−1.48,3.11)	0.62	0.49	2.46(0.36,4.55)	0.25	0.02	2.44(−0.35,5.24)	0.39	0.09
LDL-C	1.79(−0.24,3.83)	0.31	0.08	1.31(−0.56,3.18)	0.29	0.17	3.05(0.58,5.53)	0.72	0.02
TG	0.10(−3.99,4.18)	0.96	0.96	1.14(−2.97,5.26)	0.84	0.59	1.51(−3.68,6.70)	0.91	0.57

^a^ *p* value of Q-test for heterogeneity test

##### LDL-C

**Fig. 3:**
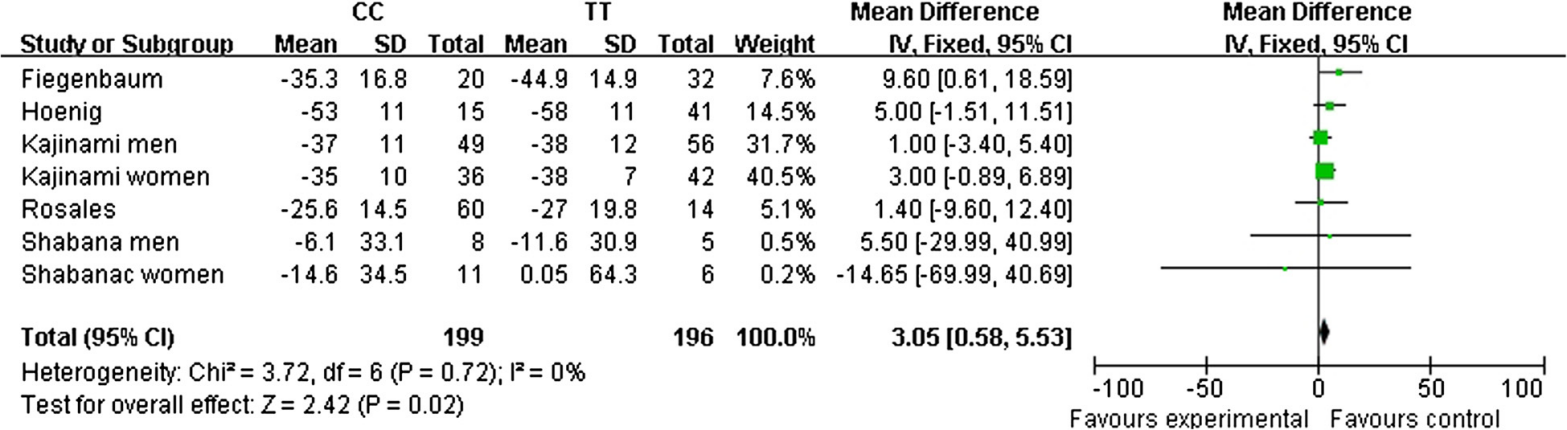
The association of the *ABCB1* C3435T polymorphism with the pooled fix-effects-based mean difference of the change in LDL-C after statin treatment. Comparison: CC vs. TT

Seven studies provided data on LDL-C levels, and the heterogeneity was low for all comparisons. The overall meta-analysis demonstrated that LDL-C lowering effects were associated with the *ABCB1* C3435T variation in homozygotes (CC vs. TT: MD, 2.29; 95 % CI, 0.37 to 4.20; *P* = 0.02; Fig. 3 and Table 3).

##### TC

**Fig. 4:**
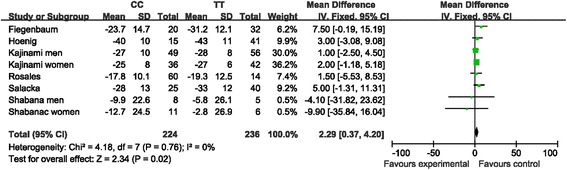
The association of the *ABCB1* C3435T polymorphism with the pooled fix-effects-based mean difference in TC levels after statin treatment. Comparison: CC vs. TT

When all eligible studies were pooled, the homozygous *ABCB1* C3435T polymorphism was correlated with decreased TC levels in patients on statin treatment (CC vs. TT: MD, 3.05; 95 % CI, 0.58 to 5.53; *P* = 0.02 Fig. 4 and Table 3). No between-study heterogeneity was detected.

##### TG

As described in Table 3, although no heterogeneity could be identified, the meta-analysis illustrated that the association between the change in serum TG after lipid-lowering therapy and the *ABCB1* C3435T variation was not significant (CC vs. CT+TT: MD, 0.1; 95 % CI, −3.99 to 4.18; *P* = 0.96; CC+CT vs. TT: MD, 1.14; 95 % CI, −2.97 to 5.26; *P* = 0.59; CC vs. TT: MD, 1.51; 95 % CI, −3.68 to 6.70; *P* = 0.57).

#### Association with myopathy

When the three eligible studies were pooled, the association between statin-related myopathy and the *ABCB1* C3435T variation was not significant. The I^2^ statistic indicated between-study heterogeneity (Table 4).

**Table 4:** The total and stratified analysis of the ABCB1 C3435T polymorphism on myopathy due to statin treatment

Myalgia	Genotype	OR (95 % CI)	P^a^	P
The total group	CC vs. CT+TT	0.69(0.19,2.53)	0.09	0.57
	CC+CT vs. TT	0.75(0.06,9.93)	0.01	0.82
	CC vs. TT	0.75(0.06,9.93)	0.01	0.82
	C vs. T	0.77(0.22,2.62)	0.002	0.67
The subgroup of more than 5 months	CC vs. CT+TT	0.33(0.11,0.98)	0.89	0.05
	CC+CT vs. TT	0.30(0.12,0.75)	0.93	0.01
	CC vs. TT	0.20(0.06,0.70)	0.55	0.01
	C vs. T	0.42(0.23,0.75)	0.94	0.004

^a^ *p* value of Q-test for heterogeneity test

The effect of the *ABCB1* C3435T polymorphism was further evaluated in a stratification analyses according to the treatment duration. The *ABCB1* C3435T polymorphism was found to be associated with a risk of myopathy in patients treated with statins more than 5 months in four models (C vs. T: OR, 0.42; 95 % CI, 0.23 to 0.75; *P* = 0.004; CC vs. TT: OR, 0.20; 95 % CI, 0.06 to 0.70; *P* = 0.01; CC+CT vs. TT: OR, 0.30; 95 % CI, 0.12 to 0.75; *P* = 0.01; CC vs. CT+TT: OR, 0.33; 95 % CI, 0.11 to 0.98; *P* = 0.05). Meanwhile, the between-study heterogeneity in the subgroup was decreased (Table 4).

### Test of heterogeneity

No significant heterogeneity was observed in three genetic comparisons with respect to HDL, LDL, TC, or TG. However, significant heterogeneity was present in all the four genetic models of the association of *ABCB1* C3435T (Table 4) with myopathy. We evaluated the source of heterogeneity based on the treatment protocol. When we stratified the trials by treatment duration, the heterogeneity was not obvious in the subgroup treated for more than 5 months (Table 4).

### Sensitivity analysis

Sensitivity analysis was performed in the genetic model producing the positive results. For the lipid-lowering comparisons, the significance of the pooled MDs was not significantly affected by the omission of the individual studies with the exception of one study [10] in the TC and LDL groups as well as in the female subgroup of Kajinami’s study [9] in the LDL and HDL-C group. Given the limited number of studies on statin-associated myopathy, we were unable to perform the sensitivity analysis in this section.

### Publication bias

**Fig. 5:**
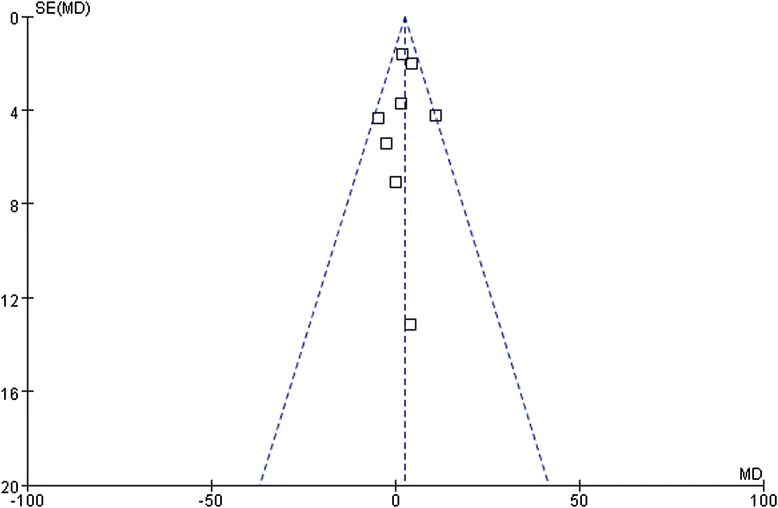
Funnel plots of the meta-analysis of the relationship between the *ABCB1* C3435T polymorphism and HDL-C change upon statin treatment. Comparison: CC vs. CT+TT

**Fig. 6:**
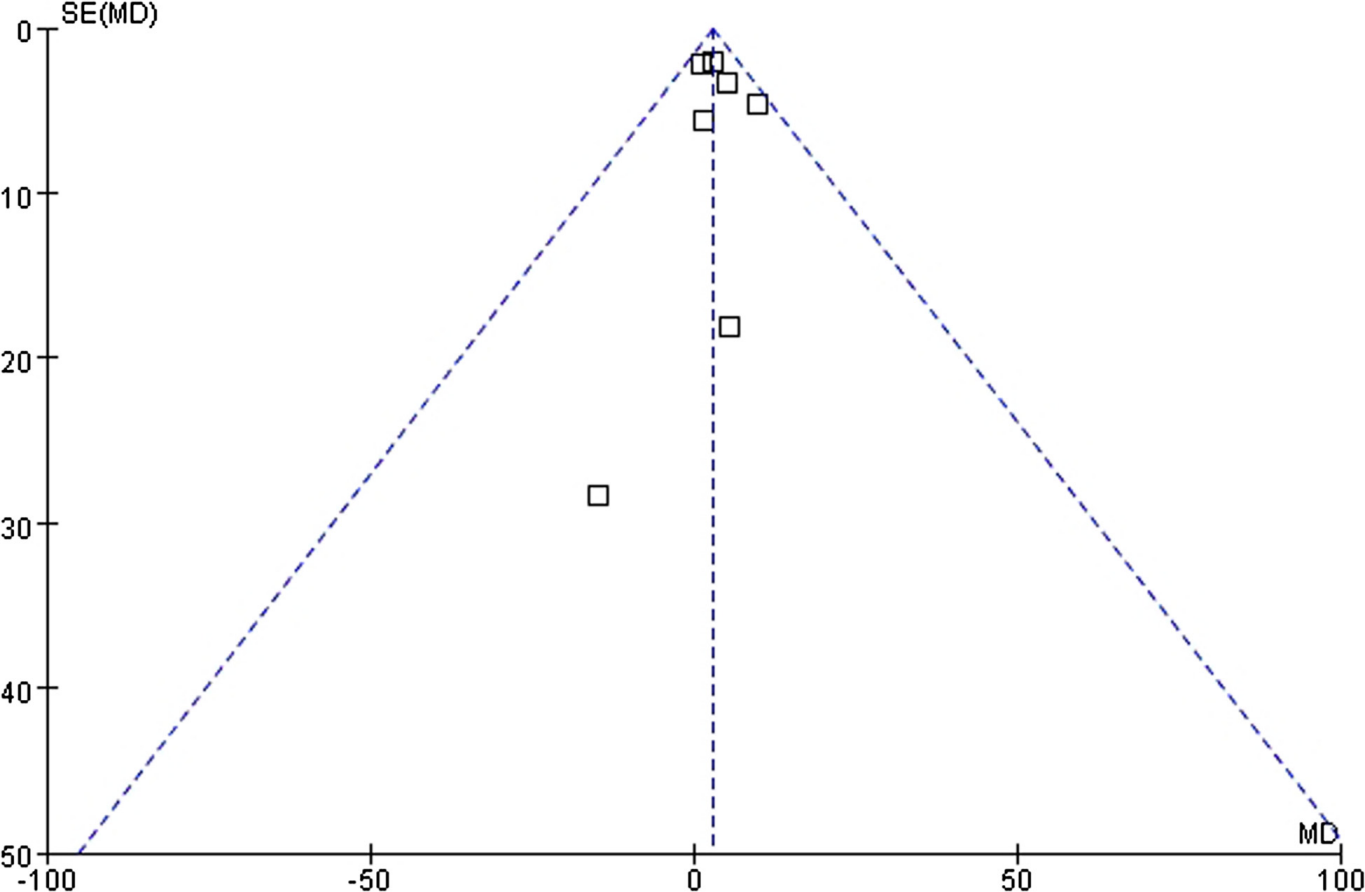
Funnel plots of the meta-analysis of the relationship between the *ABCB1* C3435T polymorphism and LDL-C change upon statin treatment. Comparison: CC vs. TT

**Fig. 7:**
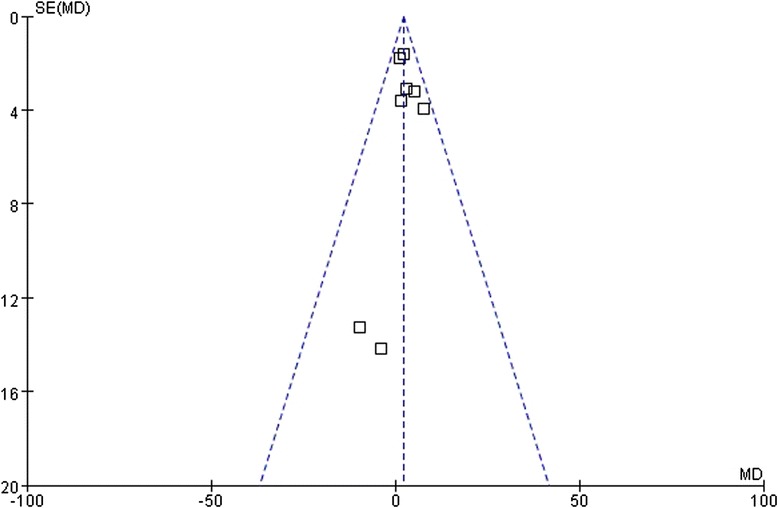
Funnel plots of the meta-analysis of the relationship between the *ABCB1* C3435T polymorphism and TC level change upon statin treatment. Comparison: CC vs. TT

A funnel plot was created to assess the publication bias of our included studies. As indicated in Figs. 5, 6 and 7, the asymmetry was not statistically significant. Therefore, no significant publication bias was detected.

## Discussion

Cholesterol-lowering therapy is a keystone in the primary and secondary prevention of CVD [22]. Statins, which are HMG-CoA reductase inhibitors, are used to improve the lipid profile in dyslipidemic patients. The *ABCB1* gene is only involved in cellular drug excretion [23] and the absorption of some drugs (for example clopidogrel [24]) but also possesses other functions: cholesterol redistribution [25]; intestinal cholesterol re-absorption [26]; regulation of cholesterol cellular trafficking; and cholesterol redistribution in cholesterol-rich microdomains of the cell membrane [27, 28]. Hence, the *ABCB1* gene and its polymorphisms may play vital roles in lipid-lowering response of statins. On the basis of varying response in the lipid-lowering efficacy and safety profiles, several genetic studies have been performed to evaluate the association between the *ABCB1* C3435T polymorphism and the lipid-lowering response in patients on statin treatment, but the results are inconclusive. There is no published GWAS studying the association examined in this meta-analysis. A former meta-analysis [29] reported that there were no significant differences in the efficacy of statin therapy between the “CC” and “CT+TT” groups.

In the present meta-analysis, we included the latest research and conducted the study more meticulously than past studies, including different genetic models and more detailed data that the former meta-analysis had not collected; our analysis produced some significant results. First, this meta-analysis indicated that the reduction in LDL-C and TC levels was associated with the *ABCB1* C3435T variation in homozygotes. Second, individuals of genotype CC+CT were more likely to exhibit an elevation in serum HDL-C upon statin treatment. However, no significant association in the TG group and other genetic models was detected with respect to the above serum lipid parameters. The results are inconsistent with those of the previous study. Although the between-study heterogeneity in the above comparison was very limited, it should also be noted. In addition to genetic polymorphisms, many factors can inference the results of this research, including the following: differences in ethnicity, age, gender, genotyping method, study period, the primary clinical characteristics, and the treatment protocol (the type of statin, the dose, and the treatment duration). Despite the best effort to perform subgroup analyses according to diverse variables, no significant outcome was identified. Recently, an investigation reported that rosuvastatin (20 mg/day) is more effective with respect to the ability to increase HDL-C levels than atorvastatin (80 mg/day) in patients with ST elevation myocardial infarction (STEMI) [30]. Hence, because of the variables listed above, the association might have been biased.

Despite the proven efficacy, large inter-individual variability exists in the risk of adverse effects in patients on statins. Muscle toxicity is a relatively common adverse effect, occurring in 1–5 % of cases [31, 32]. Severe muscle toxicity is rare but represents a significant source of mortality [33]. This toxicity sometime appears to be dose-dependent, with higher levels of statins conferring higher overall risk. Moreover, clinicians should take into consideration a series of other potential risk factors (i.e., female, advanced age, diabetes mellitus, hypothyroidism and vitamin D deficiency) [34]. In our research, by different treatment protocol, three studies with myopathy are included. This was the first meta-analysis on the relationship between the polymorphism and statin-associated muscle toxicity. The four models of polymorphism were all associated with the risk of muscle toxicity when patients were treated with statins for more than 5 months. Thus, the treatment duration may influence the safety of statin treatment. Given that limited studies were included, the results should be interpreted with caution, and further larger studies should be performed.

Statins reduce the risk of major vascular events in a linear fashion, with a 20 % risk reduction for every 1 mmol/L decrease in low-density-lipoprotein cholesterol(LDL-C) [35]. Although an association exists between the *ABCB1* C3435T polymorphism and cholesterol-lowering therapy, more attention has been paid to the interaction between SNPs and other factors. Numerous basic pharmacogenomics studies have revealed that candidate genes, such as *CETP, HMGCR, SLCO1B1, ABCB1,* and *PCSK9*,and their polymorphisms may represent pharmacogenomics biomarkers for predicting statin treatment outcomes [8]. One study evaluated the effect of the *ABCB1* haplotypes (1236C>T, 2677G>A/T and 3435C>T) on TC and LDL-C responses to simvastatin and demonstrated a reduction in the T–non-G–T haplotype frequency in patients with myalgia compared with the non-ADR group (*P* = 03) [10]. Thus, various SNPs coexisting in the same or different genes might be related to cholesterol-lowering therapy while on statin therapy, and attention should be paid to gene-gene interactions, including *SLCO1B1* or *PCSK9* with *ABCB1* C3435T.

Both genetic and environmental factors influence the lipid-lowering effect of statins. For example, smokers and patients with hypertension exhibit smaller decreases in LDL-C in response to statin treatment [36]. Furthermore, some studies have demonstrated that statins may be less effective in HIV-infected individuals, possibly as a result of their inherent viral resistance to statins or to highly active antiretroviral therapy [37]. Furthermore, drugs, such as amiodarone [38], and inflammation [39] may lead to a poor response to statin due to the decline in LDL-R protein [40]. In this meta-analysis, owing to the small population size as well as the inconsistent stratification in environmental exposures, further detection of the gene-environment interaction could not be performed. Therefore, more sophisticated gene-gene and gene-environment interactions should be included in a future analysis to obtain a more comprehensive understanding.

If a patient is highly resistant or intolerant to statin treatment, especially for those with genetic susceptibility (e.g., those with certain *ABCB1* genotypes), there are several other treatment possibilities. One such drug is ezetimibe, which is a selective inhibitor of dietary and biliary cholesterol absorption at the brush border of the intestine acting that targetsNPC1L1 [41]. The study IMPROVE-IT evaluated the potential effect of ezetimibe on major cardiovascular (CV) events and reported a clear benefit from combination treatment with simvastatin and ezetimibe in patients with acute coronary syndrome and low LDL-C [42]. Furthermore, researchers have recently begun to focus on another type of lipid-lowering drug based on *PCSK9* (proprotein convertase subtilisin-kexin type 9) inhibition by a monoclonal antibody, such as alirocumab and evolocumab; phase III clinical trials have recently been initiated. Two recent studies demonstrated that alirocumab or evolocumab, when added to statin therapy, significantly reduced LDL-C levels and reduced the incidence of cardiovascular events [43, 44]. PCSK9 inhibition may represent an option for patients who are statin-intolerant or whose LDL‑C level remains high despite maximal statin therapy with less adverse effects [45]. The above treatment options may be well suited for patients who respond poorly to statins owing to their *ABCB1* genotypes and provide fresh insight into the lipid-lowering treatment; however, substantial trails should be arranged for further assessment.

Although considerable efforts were put forth in our study design, there were some inherent limitations. First, the number of studies included is limited, especially with respect to the information on the risk of myopathy in patients treated with statin. Thus, the conclusion should be interpreted with caution. Moreover, some detailed information such as the age and sex of the patients were not available in some studies, and the studies primarily focused on patients in Latin America, all of which further limit our assessment. Furthermore, gene-gene or gene-environment interactions, can influence the clinical characteristics, and genotyping methods may affect the results. The above elements can be controlled more effectively through a separate analysis of distinct variables to which we did not have access. And we also need to promote the “clinical” value of the report widely since limited hospital has carried out the genotyping methods.

## Conclusion

In summary, this meta-analysis indicated that decreases in LDL-C as well as TC levels upon statin treatment were associated with the *ABCB1* C3435T homozygous variation, and people with the CC+CT genotype were more likely to exhibit elevated serum HDL-C. In addition, statin treatment for more than 5 months increased the risk of muscle toxicity. However, additional studies are needed to address an advanced empirical approach, and standardized stratification in further tasks is warranted to validate our findings.
